# Quantifying Social Influence in an Online Cultural Market

**DOI:** 10.1371/journal.pone.0033785

**Published:** 2012-05-09

**Authors:** Coco Krumme, Manuel Cebrian, Galen Pickard, Sandy Pentland

**Affiliations:** Media Lab, Massachusetts Institute of Technology, Cambridge, Massachusetts, United States of America; University of Oxford, United Kingdom

## Abstract

We revisit experimental data from an online cultural market in which 14,000 users interact to download songs, and develop a simple model that can explain seemingly complex outcomes. Our results suggest that individual behavior is characterized by a two-step process–the decision to sample and the decision to download a song. Contrary to conventional wisdom, social influence is material to the first step only. The model also identifies the role of placement in mediating social signals, and suggests that in this market with anonymous feedback cues, social influence serves an informational rather than normative role.

## Introduction

Predicting the next blockbuster movie can be good sport. But it’s hardly science: sleeper films can unexpectedly work their way to the top, and books ignored by critics suddenly reach best-seller status [1]. In spite of post-hoc claims that word-of-mouth marketing or classic positioning “made” a given product, predicting the success of songs, books, and movies remains largely a voodoo science [2].

A recent experimental study [3] found that the addition of social influence to a cultural market increased the unpredictability as well as the inequality of the market share of individual products. However, the study did not propose a model to describe how such social forces might operate. Here, we present a parsimonious model that sheds light on social behavior in this market. Our model does not rely on assumptions about heterogeneous preferences [4] or solely on the generic notion of herd behavior [5] to explain the outcomes of anonymous social influence: rather, we treat social influence as a variable whose effect grows as the market matures.

MusicLab is an online laboratory created in 2004 to evaluate experimentally the role of social influence in the success of cultural products. Researchers invited consumers (about 14,000 in total) to sample 48 previously unknown pop songs via a website, to rate them, and to download whichever of the songs they liked. Songs were arranged on the screen in either a 16×3 grid (Experiment 1) or a single column (Experiment 2).

In each experiment, each visitor was assigned randomly to one of two conditions. In the social influence condition, of which there were eight instances or “worlds”, participants received additional information about the number of times each song had been downloaded by his peers, and songs in Experiment 2 were ordered on the screen according to past download count. Songs were shown in random order in the independent condition [1], [6], [7].

Results from the MusicLab experiments suggest that, in this market, information about the behavior of others contributes to greater inequality (differential market share) and unpredictability (variance of possible outcomes), compared to the inequality and unpredictability in the non-social condition.

While Salganik et al. report empirical findings, they do not describe a mechanism for the process of social influence. Others have subsequently proposed theoretical models to explain how a set of individual preferences and responses can create such outcomes. Borghesi and Bouchard model each participant’s decision as a multiple-choice situation, and two conditions of “weak” and “strong” herding that fit the empirical data [8]. Hendricks et al develop an equilibrium model to explain how an “anonymous” non-differentiated herd affects low versus high quality products [9]. Our approach differs in several regards. First, we observe the progression of inequality and unpredictability over the course of each experiment, and to compare it to simulation results. Unlike Borghesi and Bouchard, we do not consider decision-making a multiple-choice situation: we model independent listens rather than listeners, where a listen occurs according to a by-song probability derived from its appeal and a coefficient for social forces.

Social influence exists in non-experimental markets as well, in the form of herding and informational cascades [5] as well as individual decision-making in the presence of complex information [10], [11]. Of course, real markets offer a host of complexities intentionally omitted from the MusicLab experiment in order to test the researchers’ hypothesis, such as the possibility for stronger, peer-to-peer social influence and external marketing forces [12], [13]. We discuss below some of the ways in which the experimental setting both resembles and differs from real-world markets. Here, our focus is on parsimoniously modeling the social dynamics of the MusicLab marketplace itself.

To do so, we develop a model for the empirical results that distinguishes between a song’s quality and the signal generated by the visible downloads. From the empirical data, we observe that song selection can be modeled as a sequential process in which each song has a probability of being sampled, independent of the other songs a listener chooses, and then an independent probability of being downloaded. Modeling choices are based on empirical observations of user behavior in this market. We describe this process and the model inputs in detail in the following sections.

## Results

In the MusicLab experiment, the authors record the choices of participants who enter the market one-by-one. Here, we model *song listens* rather than market participants, and validate this approximation by examining the consistency of sampling across different participants’ propensity to sample more or fewer songs. We find that people who listen to a total of *n* (where n<40) songs in the system have, on average, the same probability of sampling a particular song *i*. In fact, over the entire population, the *probability song i will be sampled* does not depend on *the distribution of volume of listens* in the population who samples it (r-squared = 0.1, Figure S1]. Additionally, the conditional probability of downloading a song (given it was sampled) does not depend on the total number of songs a participant samples [Figure S2, Figure S3].

**Figure 1:**
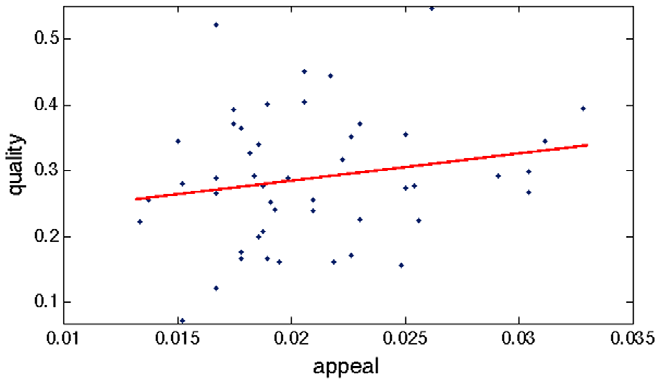
Quality and appeal are independent. Values are shown for quality and appeal corresponding to the 48 songs in Experiment 2, independent condition. *R* ^2^ = 0.012.

Again from the empirical data, we observe that there are two stages of decision-making, listening and downloading, that occur according to fixed but independent distributions. The result of the second step (downloading), but not the first (listening), is ultimately visible to future market entrants. We observe that the probability that a user clicks on a song (which we ascribe to the *appeal* of the song's title) is independent of the conditional probability he downloads the same song, given he listened to it (which we call the song's *quality*) [Figure 1]. This finding suggests that in this market, the perception of quality is not subject to social influence.

Sociologists distinguish between the normative and informational facets of social influence [14]: while the former might compel a person to do as others do, the latter acts as a signal of what others like. Because song appeal and propensity to download are independent, we assert that social influence works as a purely informational force in this market (in other markets, of course, normative influence may be much more relevant).

**Figure 2:**
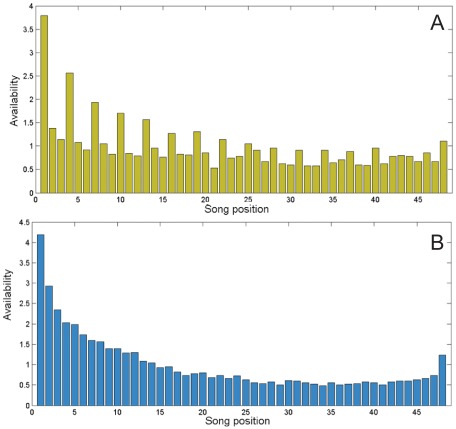
Availability in the independent world of Experiments 1 (A, top) and 2 (B, bottom), indexed to 1. The availability of a position n describes the likelihood that a song in that position will be sampled (where n = 1 is the top left corner in Experiment 1, and the topmost position in Experiment 2, and n = 48 is the bottom right corner in Experiment 1 and the bottom of the column in Experiment 2). Availability serves as a multiplier in calculating the total probability of a song being sampled, given its position-independent appeal, and its position at a given time in the market. In Experiment 1 (Figure 2a), songs on the left side of the grid are more likely to be sampled, all else equal, than songs on the right. In Experiment 2 (Figure 2b), songs at the top of the column, as well as the final song, are more likely to be sampled.

In both experiments, a song’s appeal depends on two factors: first, the inherent attractiveness of its title, and second, its positioning on the screen, which we call *availability*. Availability is defined as the probability that a song *i* will be sampled in a given position *p*:
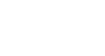
We find that in MusicLab, positioning matters [Figure 2]: in Experiment 2, participants are more likely than random to click on songs at the top of the list than on those mid-way down. In Experiment 1, the grid interface, the general trend is the same, with a small spike in multiples of three, representing songs positioned on the left side of the screen.

A song’s *appeal* reflects the probability that a participant will want to sample or try it. We can think of appeal as a function of the final listen counts in the independent condition, where:
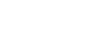
for *k* = songs 1 through 48. Here, appeal simply represents the probability of sampling a song, due the attractiveness of the song title in each of the social worlds.

Quality, in turns, measures the conditional probability of download, which we derive directly from the independent world for each of Experiments 1 and 2:
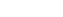
Finally, we find the total run length of each experimental world, as well as the total download count, which round to an average of 2700 listens in Experiment 1, 2500 listens in Experiment 2, and 1000 downloads in the social conditions of both experiments (with slightly higher variance in total downloads across the eight worlds of Experiment 1).

### Model Description: Pólya Urn

**Figure 3:**
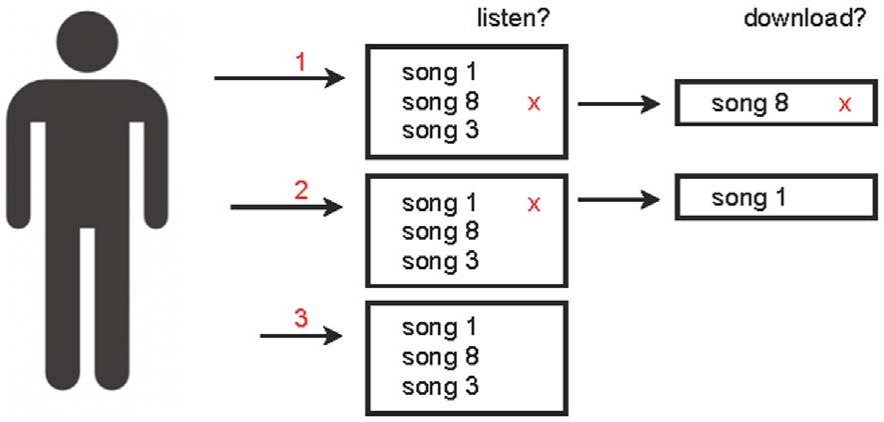
Song selection as a two-step process. A listen first selects which song(s) he will listen to, and after listening, decides whether or not to download the song. The first decision is made based on the appeal of a song; the second based on its quality. If a listener listens to more than one song, this process is repeated.

Using these inputs, we model the dynamic download count *D_i_* of each song *i* over time, and use the final download counts to compute inequality and unpredictability. The model consists of two steps for each entrance of a listener to the market [Figure 3]. These steps are repeated if a listener elects to try more than one song:

1. Select a song to sample, based on its appeal, position, and current download count
2. Choose whether or not to download the song, based on its quality

In the first step, a participant enters the market and chooses a song at random to sample based on a combination of its appeal *A_i_* the availability score of its current position *_Vi,t_*, and its current download count *D_i,t_*.

The probability that song *i* is sampled is 
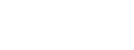


Here is a scaling factor, constant across all songs, which captures the strength of the social signal. As *D_i_* grows over the course of the experiment, its value contributes increasingly to the probability a song will be sampled.

In the second step, the user downloads the chosen song with probability *q_i_*, and *D_i_* is incremented if a download occurs.

While we model *listens* rather than individuals with different listener types, this assumption has little effect on model outcomes [Figure S2]. In other words, our model can be said to describe one listener at a time, who listens to at least one song, after which he can choose to repeat these steps, up to a total of 48 times, or to exit the market by not selecting a song.

The decision to listen to a song leaves no signal for others: a song's listen count is invisible to other participants. By contrast, download count is seen by users in the social influence condition (but not by those in the independent condition). So, a user arriving late to the market with social signal receives more information about the songs chosen for download by his peers than does an earlier entrant.

### Inequality and Unpredictability

In the original experiment, inequality is defined by the Gini coefficient,
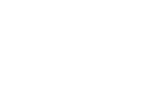
where *D_i_* is the final download count, or market share, of song *i*, and *S* the total number of songs.

Unpredictability is measured across multiple worlds, with the unpredictability for song *i*

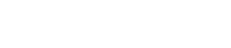
where *m_i,j_* is the market share of song *i* in world *j* and total unpredictability 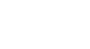


Using the sets of inputs for Experiments 1 and 2, we simulate eight social influence worlds of 2700 listens in Experiment 1, and 2500 listens in Experiment 2 (with resulting download counts ranging from 900–1100) and compute the resulting inequality and unpredictability. To calculate these values for the independent conditions, we run the simulation without the effect of the visibly increasing download count (and its concomitant social effects), so that the probability of sampling song *i* is simply
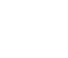



**Figure 4:**
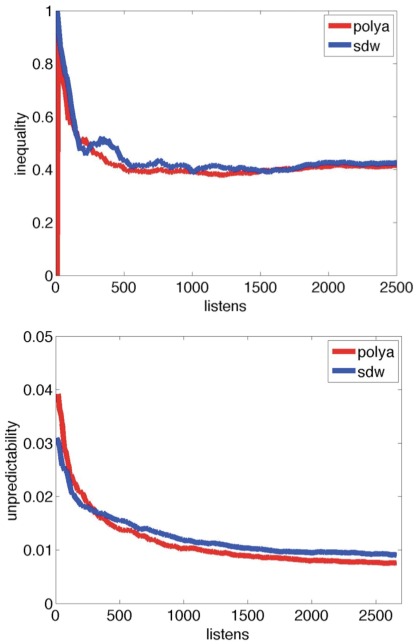
Inequality (top) and unpredictability (bottom) over the course of the market, with alpha = 900. Inequality is shown for Experiment 1, world 3. RMSE of simulated market’s unpredictability is = 0.0017, and average of inequality is = 0.093.

**Figure 5:**
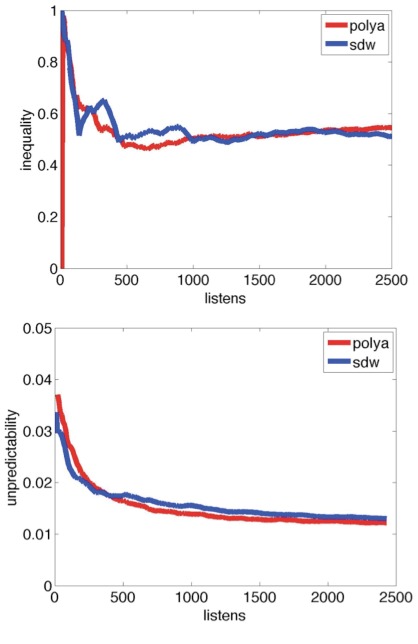
Inequality (top) and unpredictability (bottom) over the course of the market, with alpha = 200. Inequality is shown for Experiment 2, world 5. RMSE of simulated market’s unpredictability is = 0.0012, and average of inequality is = 0.0516.

For each experiment, we find, through simulation, the value of alpha that offers the best fit for the values of unpredictability and inequality observed in the original experiment [Table 1]. We are able to replicate the values of inequality and unpredictability over the course of both experiments [Figure 4, Figure 5, Figure S4].

**Table 1:** Values of unpredictability and inequality from simulated (polya) and original (SDW) markets.

Experiment 1, alpha = 900		
	Polya	SDW
Inequality (mean)	0.4	0.35
Inequality NS	0.28	0.25
Unpredictability	0.0072	0.008
Unpredictability NS	0.0053	0.005**
**Experiment 2, alpha = 200**		
Inequality (mean)	0.52	0.51
Inequality NS	0.22	0.2
Unpredictability	0.012	0.0127
Unpredictability NS	0.005	0.004**

* NS = non-social

** averaged from sub-populations

The inequality (measured by Gini) is a mean of the results of 8 worlds. In the original experiments, the value for non-social unpredictability was determined by taking subpopulation of the independent condition. The simulated value for unpredictability is determined from 8 simulated non-social worlds.

We observe a substantially higher alpha in Experiment 1 (songs displayed in a grid) versus Experiment 2 (songs displayed in a column), suggesting that the impact of a song’s appeal is more important in the early stages of the market of Experiment 1. This could be due to the fact that all songs are visible on a single grid, and there is no need to scroll down a long list: a listener employs social information differently to make his choice, compared to the column layout of Experiment 2.

With a frugal model that parallels the decision-making process of the listener (who elects to sample a song based on its inherent appeal, its screen position, and how many others have downloaded it; then decides whether to download it based on its quality), we are able to reproduce the results of the original Experiment 2 with RMSE = 0.0012 for unpredictability and 0.0516 for inequality over the course of the market, and for Experiment 1, RMSE = 0.0017 for unpredictability and 0.093 for inequality.

To summarize the findings described thus far, we first determined, from the experimental data, that the perception of quality, which drives the propensity to download, is not influenced by social forces in this market. Second, with a single scaling factor, we were able to simulate results for inequality and unpredictability over the course of the experiment, suggesting that the dynamics of the market are one of an increasing impact of social factors as the experiment progresses. That is, over time, the weight of the download count grows relative to the appeal of songs in determining a listener’s choice of music to sample. Finally, the positioning of songs has an impact, and in particular the screen layouts of Experiments 1 and 2 yield different scaling factors, suggesting that the way in which products are positioned impacts the magnitude of the social forces.

### Long-run Dynamics

In the short run, sampling in the MusicLab market is based largely on initial screen position and on the appeal of songs’ titles.

In the longer run, in our model the download to listen ratio increases, suggesting that a larger proportion of higher quality songs are being sampled. Simulating 100,000 listens, the download count to listen count ratio rises significantly, to about 51 downloads per 100 listens in Experiment 2 (in the typical 2500-listen world, this ratio hovers around 39 downloads per listen). Because the number of listens is fixed in the simulation, the higher ratio indicates that a greater number of songs are being downloaded (and that higher quality songs are being sampled more frequently). Of course, in a real market, users may adjust their behavior as market conditions change: for example, they may sample more or fewer songs than earlier entrants.

When social influence is present, unpredictability sinks slightly (to a mean of .0083 with a standard deviation of .00043 on 100 runs after 100,000 listens in Experiment 2), while Gini rises (to a mean of 0.69 with standard deviation 0.033). The unpredictability of the non-social worlds declines significantly (after 100,000 listens in Experiment 2, it reaches a mean of .00005, or about 1% of its value at 2500 listens).

### Markets with Social Influence

Of course, real markets differ from the MusicLab experiment in several regards. It is unlikely that a real market would reach even several thousand listens in isolation. Exogenous marketing cues can push behavior in significant ways, as can the non-independence of quality and appeal, such as in the case of disproportionate advertising resources devoted to books anticipated to have high quality [2].

Moreover, real markets typically see a greater volume of products, as well as the dynamic entry of new products to the system. A person cannot view every film or read every book published (nor, in many cases, does he have the chance to sample such products before buying), so he relies to some extent on social signals as filters. It is likely that these real-world cues are a mixture of informational (“all of colleagues have read it, so I should give it a try”) and normative (“my best friend recommends it, so I should read it to be able to discus with her”). Normative information is further strengthened by the presence of real relationships [15], versus the simple download counts that stand in for social signals in the MusicLab marketplace.

Yet in a number of markets, such as online retail of goods in the “long tail”, marketing plays a lesser role [16], and the actions of buyers can influence how the seller promotes his products, and thus how future entrants make their decisions. In the MusicLab experiment, we are able to observe the outcomes created by social feedback and incremented downloads alone. In the presence of social-based sorting, the outcome of the market becomes less certain.

## Discussion

Our model is able to account for the empirical outcomes of the MusicLab experiment given market size, distribution of appeal, availability and quality, and strength of the social signal. Future work might build on these results, by extending such a model to include the dynamic entry of songs to the market, non-homogenous listeners (e.g. agents with different preferences over genres), and variants on the social signal.

Although the market in question was created for the purpose of experimentation, it represents thousands of real people expressing live preferences about songs. Such controlled web-based experiments can offer insight into how social forces impact markets. Here, we develop a parsimonious model that explains observed data, and show that social influence serves a largely informational role in the present market. The behavior of others impacts what an individual will try, but has only indirect effect on what he buys.

Finally, we argue that the missing link in previous analysis of this data is the effect of availability: aggregate opinion is important insofar as it helps to modulate the salience of a song to new users entering the system. Product positioning and social media are often considered separately in marketing strategy: in this market, the two are closely linked. After all, if we’re to believe Woody Allen, 80% of success is showing up.

## Supporting Information

Figure S1 **In our model, we consider** ***song listens*** **rather than individual** ***listeners*** **.** We validate this approximation by examining the consistency of sampling across different participants types, where a user’s type is defined by his propensity to sample more or fewer songs. We find that users who listen to a total of *n* (where n<40) songs in the system have, on average, the same probability of sampling a particular song *i*. In other words, over the entire population, the *probability song i will be sampled* does not depend on *the distribution of volume of listens* in the population who samples it. Figure S1 shows, for each listener type on the vertical axis, the total distribution of songs sampled by listeners of that type in the independent condition (here, Experiment 2), ranging from blue (min) to red (max). The y-axis describes listener types: with each row representing a type, from users who listened to a single song at the top to those who elected all 48 at the bottom. The x-axis represents the 48 distinct songs, organized alphanumerically as in the original experiment (see 2006 paper for song names), from left to right. No listener listened to precisely 32, 34, or between 39–41 songs, and listeners who listened to 48 songs (clearly) sampled every song. Listeners of different types spread their listens between songs approximately evenly (ANOVA p << 0.01).
(TIFF)

Figure S2 **The conditional probability of downloading a song (given it was sampled) does not depend on the total number of songs a participant samples.** Figure S2 shows the average conditional probability of download versus listener type.
(TIFF)

Figure S3 **Listener type on the vertical axis, versus conditional probability of download.**
(TIFF)

Figure S4 **Inequality results in each of the 8 worlds of Experiments 1 and 2.**
(TIFF)
