# Supplementary figures and images for: Quantifying Social Influence in an Online Cultural Market
Source: PLoS One. 2012 May 9;7(5):e33785. doi: 10.1371/journal.pone.0033785 (PMC3348939; doi:10.1371/journal.pone.0033785)

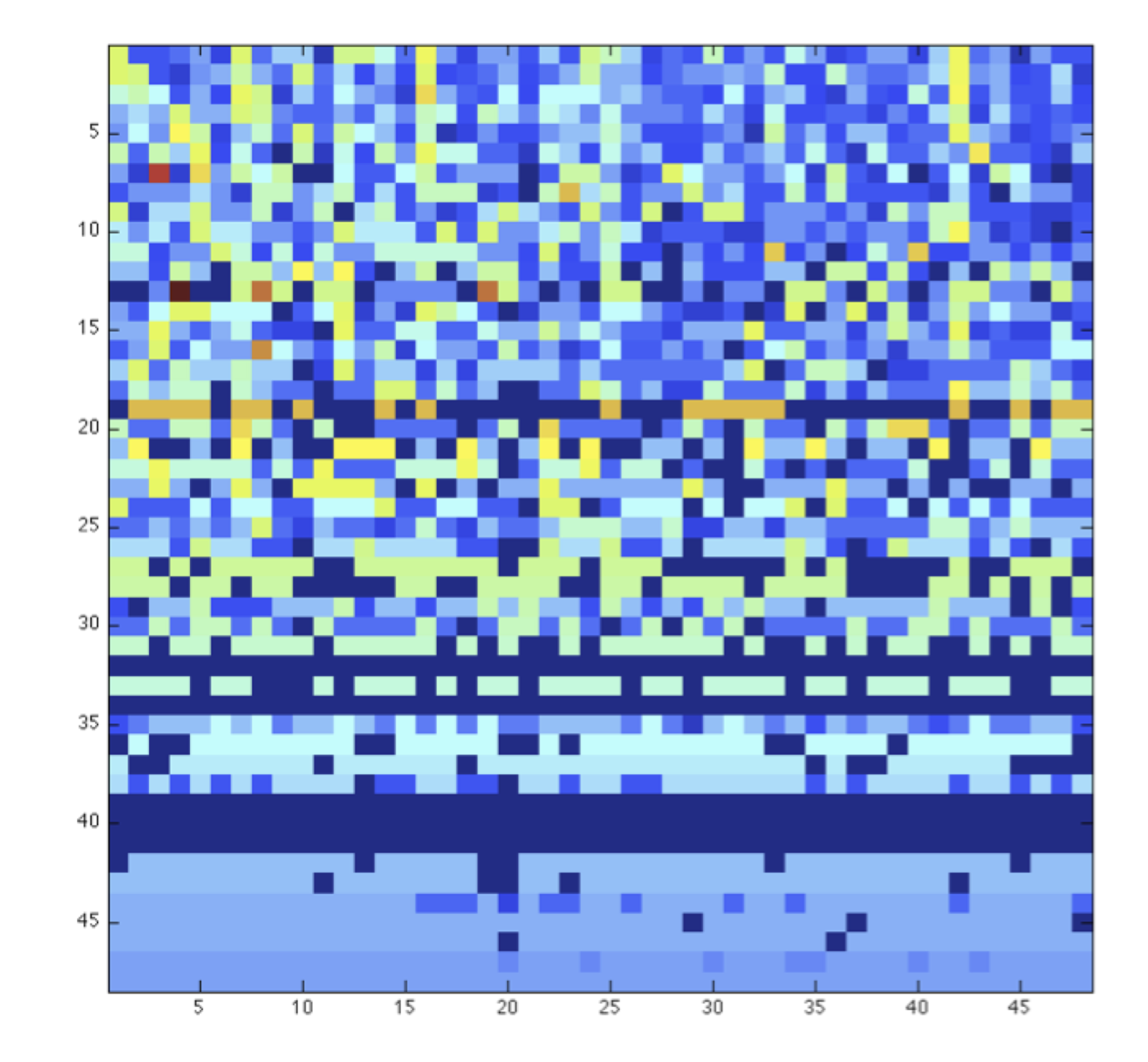

Supplement: Figure S1 — In our model, we consider song listens rather than individual listeners . We validate this approximation by examining the consistency of sampling across different participants types, where a user’s type is defined by his propensity to sample more or fewer songs. We find that users who listen to a total of n (where n<40) songs in the system have, on average, the same probability of sampling a particular song i. In other words, over the entire population, the probability song i will be sampled does not depend on the distribution of volume of listens in the population who samples it. Figure S1 shows, for each listener type on the vertical axis, the total distribution of songs sampled by listeners of that type in the independent condition (here, Experiment 2), ranging from blue (min) to red (max). The y-axis describes listener types: with each row representing a type, from users who listened to a single song at the top to those who elected all 48 at the bottom. The x-axis represents the 48 distinct songs, organized alphanumerically as in the original experiment (see 2006 paper for song names), from left to right. No listener listened to precisely 32, 34, or between 39–41 songs, and listeners who listened to 48 songs (clearly) sampled every song. Listeners of different types spread their listens between songs approximately evenly (ANOVA p << 0.01). (TIFF) [file pone.0033785.s001.tiff]

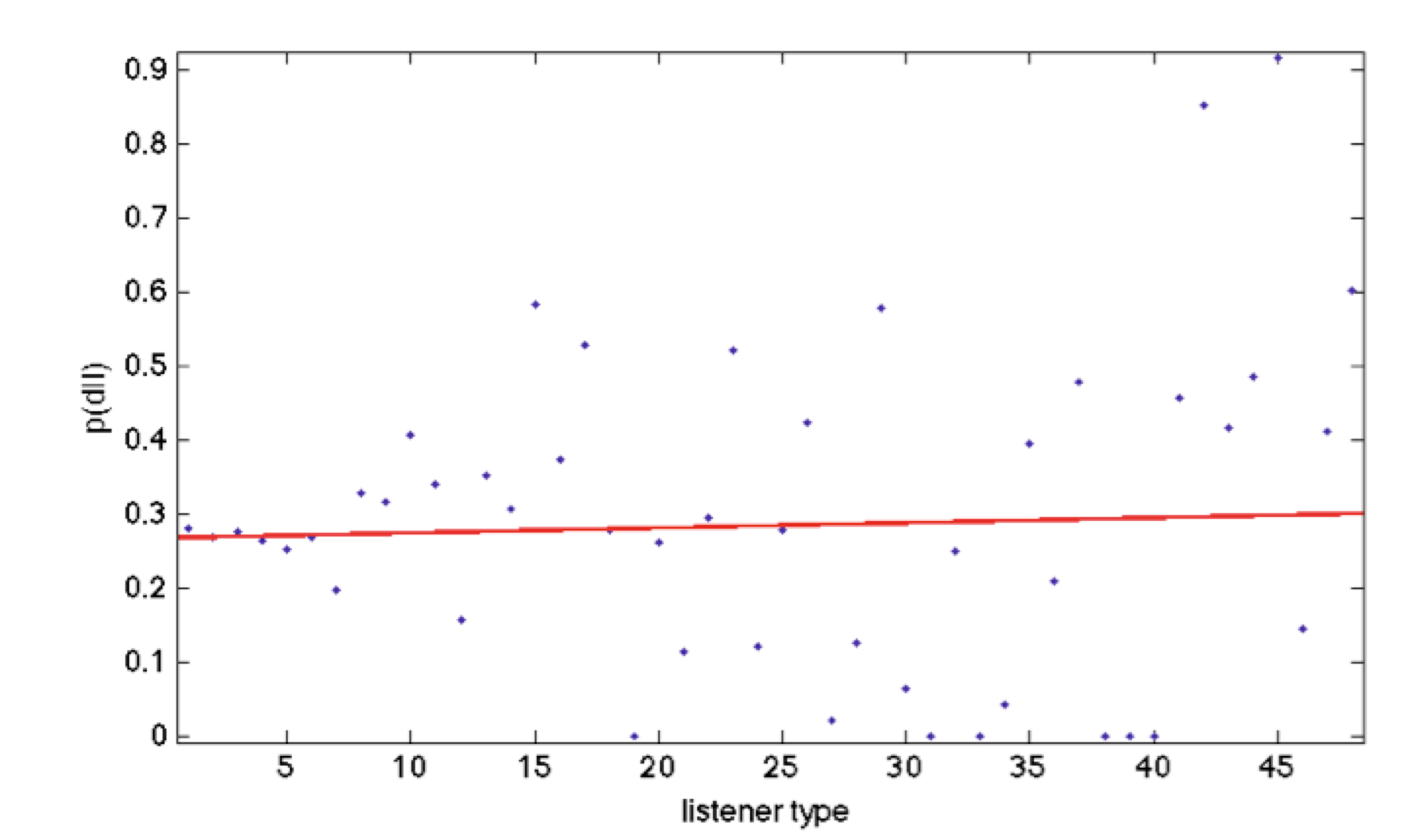

Supplement: Figure S2 — The conditional probability of downloading a song (given it was sampled) does not depend on the total number of songs a participant samples. Figure S2 shows the average conditional probability of download versus listener type. (TIFF) [file pone.0033785.s002.tiff]

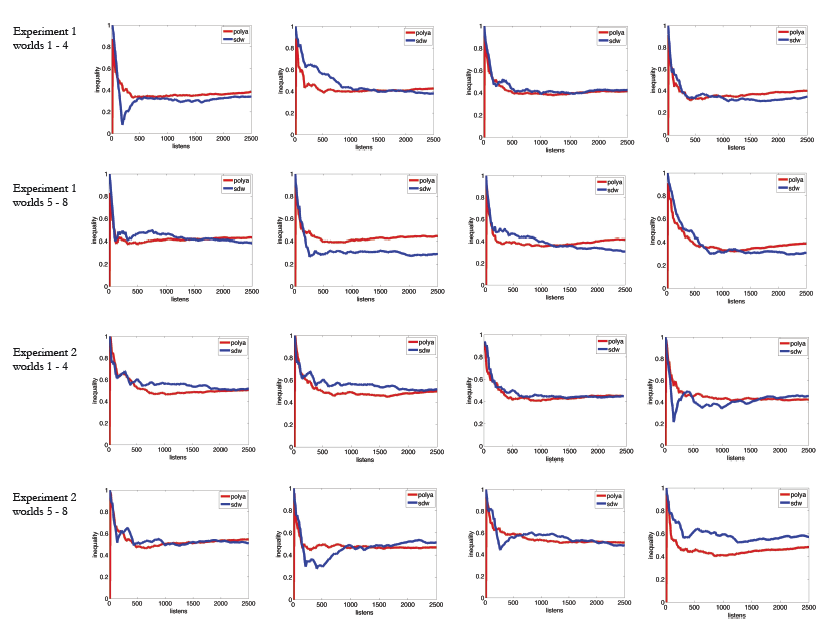

Supplement: Figure S4 — Inequality results in each of the 8 worlds of Experiments 1 and 2. (TIFF) [file pone.0033785.s004.tiff]
